# Noninvasive Prenatal Molecular Karyotyping from Maternal Plasma

**DOI:** 10.1371/journal.pone.0060968

**Published:** 2013-04-17

**Authors:** Stephanie C. Y. Yu, Peiyong Jiang, Kwong W. Choy, Kwan Chee Allen Chan, Hye-Sung Won, Wing C. Leung, Elizabeth T. Lau, Mary H. Y. Tang, Tak Y. Leung, Yuk Ming Dennis Lo, Rossa W. K. Chiu

**Affiliations:** 1 Centre for Research into Circulating Fetal Nucleic Acids, Li Ka Shing Institute of Health Sciences, The Chinese University of Hong Kong, Shatin, New Territories, Hong Kong SAR, China; 2 Department of Chemical Pathology, The Chinese University of Hong Kong, Prince of Wales Hospital, Shatin, New Territories, Hong Kong SAR, China; 3 Department of Obstetrics and Gynaecology, The Chinese University of Hong Kong, Prince of Wales Hospital, Shatin, New Territories, Hong Kong SAR, China; 4 Department of Obstetrics and Gynecology, University of Ulsan College of Medicine, Asan Medical Center, Seoul, Korea; 5 Kwong Wah Hospital, Kowloon, Hong Kong SAR, China; 6 Tsan Yuk Hospital, Department of Obstetrics and Gynaecology, University of Hong Kong, Hong Kong SAR, Hong Kong; Tel Aviv University, Israel

## Abstract

Fetal DNA is present in the plasma of pregnant women. Massively parallel sequencing of maternal plasma DNA has been used to detect fetal trisomies 21, 18, 13 and selected sex chromosomal aneuploidies noninvasively. Case reports describing the detection of fetal microdeletions from maternal plasma using massively parallel sequencing have been reported. However, these previous reports were either polymorphism-dependent or used statistical analyses which were confined to one or a small number of selected parts of the genome. In this report, we reported a procedure for performing noninvasive prenatal karyotyping at 3 Mb resolution across the whole genome through the massively parallel sequencing of maternal plasma DNA. This method has been used to analyze the plasma obtained from 6 cases. In three cases, fetal microdeletions have been detected successfully from maternal plasma. In two cases, fetal microduplications have been detected successfully from maternal plasma. In the remaining case, the plasma DNA sequencing result was consistent with the pregnant mother being a carrier of a microduplication. Simulation analyses were performed for determining the number of plasma DNA molecules that would need to be sequenced and aligned for enhancing the diagnostic resolution of noninvasive prenatal karyotyping to 2 Mb and 1 Mb. In conclusion, noninvasive prenatal molecular karyotyping from maternal plasma by massively parallel sequencing is feasible and would enhance the diagnostic spectrum of noninvasive prenatal testing.

## Introduction

The presence of fetal DNA in maternal plasma has opened up exciting possibilities for noninvasive prenatal testing [1], [2]. Recently, there has been much interest in the use of massively parallel sequencing (MPS) for analyzing circulating fetal DNA for prenatal testing purposes. Thus, fetal trisomies 21, 13, 18 and selected sex chromosomal aneuploidies have been detected using MPS on maternal plasma DNA [3]–[7] and have been rapidly introduced into clinical service.

Apart from abnormalities due to copy number changes involving a whole chromosome, it would be important to evaluate whether the MPS-based analysis of maternal plasma might be sensitive enough for detecting subchromosomal deletions or duplications. In this regard, Peters et al reported the detection of a 4.2 Mb deletion on chromosome 12 in a maternal plasma sample obtained at the 35^th^ week of gestation [8]. Jensen et al reported the detection of a 3 Mb deletion on chromosome 22 in maternal plasma samples obtained from two pregnant women at the 19^th^ and 20^th^ weeks of gestation [9]. Apart from the deleted region, Peters et al also performed statistical analysis on another region on chromosome 12, as well as 20 nonoverlapping 4 Mb regions on chromosome 14 [8]. Jensen et al, on the other hand, only focused their statistical analysis on the deleted region on chromosome 22 [9]. Thus, from the data presented by Peters et al and Jensen et al, it is not clear if the approach would be robust enough for a genomewide survey of microdeletions or microduplications, or indeed for the noninvasive determination of a fetal karyotype.

Lo et al reported that fetal single nucleotide polymorphisms (SNPs) can be genotyped in a genomewide scale using maternal plasma DNA sequencing [10]. In particular, these investigators have demonstrated that SNP alleles and mutations for single gene disorders that are inherited by a fetus from its mother can be elucidated by a process called relative haplotype dosage analysis [10]. Fan et al confirmed the robustness of relative haplotype dosage analysis and used this approach to detect a ∼2.85 Mb deletion inherited by a fetus from its mother [11]. There are two concerns for using this method for the clinical implementation of noninvasive prenatal karyotyping. First, this method requires maternal haplotyping to be performed which would require additional analytical steps [12], [13] or pedigree analysis. Second, it is unclear if this method could be used to detect de novo subchromosomal deletion or duplication.

We have recently shown that through the use of shotgun MPS of the plasma DNA of cancer patients, one could noninvasively obtain a ‘plasma karyotype’ of a cancer at 1 Mb resolution [14], [15]. In this report, we sought to apply a similar approach for obtaining the prenatal molecular karyotypes of a number of fetuses by shotgun MPS of maternal plasma DNA.

## Materials and Methods

### Ethical Statement

The study was approved by the Joint Chinese University of Hong Kong – Hospital Authority New Territories East Cluster Clinical Research Ethics Committee. We recruited pregnant women with written informed consent from the Prince of Wales Hospital, the Kwong Wah Hospital and the Tsan Yuk Hospital in Hong Kong, and the Asan Medical Center in Seoul.

### Sample Collection

For cases 01, 02, and 03, maternal peripheral blood samples were collected into EDTA-containing tubes after invasive procedures (Table 1). For cases 04, 05 and 06, maternal peripheral blood samples were collected before performing any invasive procedures. Maternal blood samples were drawn at 12 3/7 to 28 4/7 weeks of gestation (Table 1).

**Table 1:** Sample information.

Case no.	Gestational age at plasma collection (weeks)	Plasma sampling relative to invasive procedure	Invasive procedure	Chromosomal aberration	Methods used to confirm karyotype
01	24 1/7	Post-invasive	Cordocentesis	22q11.2 microdeletion	FISH
02	28 4/7	Post-invasive	Cordocentesis	22q11.2 microdeletion	FISH
03	22 5/7	Post-invasive	Aminocentesis	22q11.2 microdeletion	QF-PCR and FISH
04	12 3/7	Pre-invasive	Chorionic villus sampling	22q11.2 microduplication (2.4 Mb)	Array CGH
05	20 2/7	Pre-invasive	Amniocentesis	22q11.2 microduplication (2.4 Mb)	Array CGH
06	21 4/7	Pre-invasive	Aminocentesis	3q29 microduplication (5.1 Mb); 4q32.1-q35.2 microdeletion (32.9 Mb)	Array CGH

Among the six test samples, there were three cases (cases 01, 02 and 03) of fetal de novo 22q11.2 microdeletion, one case (case 04) of fetal de novo 22q11.2 microduplication (2.4 Mb) and one case (case 05) of maternally-inherited 22q11.2 microduplication (2.4 Mb). There was also one case (case 06) in which the mother had a balanced translocation of t(3;4)(q29;q32) and the fetus was found to have 3q29 microduplication (5.1 Mb) and 4q32.1-q35.2 deletion (32.9 Mb). Full karyotyping was performed and the fetal karyotypes were further ascertained by array comparative genomic hybridization (array CGH) [16], fluorescence in situ hybridization (FISH) or a combination of quantitative fluorescence PCR (QF-PCR) and FISH.

In addition, we collected a group of eight singleton pregnant cases with normal fetal karyotypes as reference controls for downstream data analysis.

### Sample Processing and DNA Extraction

Peripheral blood samples were centrifuged at 1600 g for 10 min at 4°C and the plasma portion was recentrifuged at 16000 g for 10 min at 4°C [17]. We extracted cell-free DNA from 1.8 to 8.4 mL of maternal plasma with the QIAamp DSP DNA Blood Mini Kit (Qiagen) as described previously [3]. The extracted plasma DNA was quantified by a real-time PCR assay targeting the *leptin* (*LEP*) gene as described previously [18].

### Plasma DNA Sequencing

We prepared sequencing libraries of plasma DNA with the Paired-End Sequencing Sample Preparation Kit (Illumina) as described previously [19]. Due to the variable volume of maternal plasma available, we aimed to have a relatively consistent amount of plasma DNA input for library preparation. We thus used 13 to 20 ng of the extracted plasma DNA for library preparation which corresponded to the amount extracted from 1.5 to 2.2 mL of maternal plasma. The adaptor-ligated plasma DNA was enriched by a 12-cycle PCR. We performed cluster generation on a cBot clonal amplification system (Illumina) with the TruSeq PE Cluster Generation Kit v3 (Illumina). Each library (both test and reference samples) was sequenced with one lane of a flow cell on a HiSeq 2000 sequencing system (Illumina) in a paired-end format of 50-bp×2. Sequence data have been deposited at the European Genome-Phenome Archive (EGA, http://www.ebi.ac.uk/ega/), which is hosted by the European Bioinformatics Institute (EBI), under the accession number EGAS00001000439.

### Sequence Alignment and Filtering

Paired-end reads were aligned to the non-repeat masked human reference genome (NCBI Build 36.1/hg18) using the Short Oligonucleotide Alignment Program 2 (SOAP2) (http://http://soap.genomics.org.cn/). We allowed up to two nucleotide mismatches for each member of the paired-end reads. Only paired-end reads with both ends aligned to the same chromosome with the correct orientation, spanning an insert size ≤600 bp were included in downstream analysis. We also removed duplicated reads which were defined as paired-end reads showing identical start and end positions in the human genome.

### Calculation of the Genomic Representation

We first divided each chromosome into 100-kb bins and performed locally weighted scatterplot smoothing (LOESS) to correct for GC-associated bias on the sequenced read counts [20]. All the calculations below were based on the GC-corrected read counts.

For the detection of subchromosomal aberrations, we merged the 100-kb bins into 1-Mb bins and calculated the genomic representation of each 1-Mb bin (GR_x−y_), where x and y denote the start and end genomic coordinates of the 1-Mb bin. We determined the number of sequence reads originated from each 1-Mb bin and calculated the GR_x−y_ using this equation [20]:
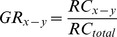
where RC_x−y_ is the read counts for the 1-Mb bin; and RC_total_ is the total read counts.

### Calculation of z-scores

We used the group of eight singleton pregnant cases with normal fetal karyotypes as the reference for the analysis of subchromosomal copy number aberrations.

We determined the mean and the standard deviation of the genomic representation of each 1-Mb bin (GR_x−y_) of the reference group and calculated the z-score for each 1-Mb bin of the test sample using this equation:
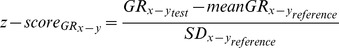
where 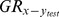 is the genomic representation of the 1-Mb bin in the test sample; mean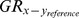 and 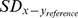
are the mean and the standard deviation of the genomic representation of the 1-Mb bin of the reference samples. To minimize the systematic inter-sample variations between different chromosomes, we performed median correction for each chromosome. Thus, the median genomic representation of all the bins on a particular chromosome was used as a baseline. For all bins located on that particular chromosome, the difference from this baseline value was used for the calculation of the z-score.

### Calculation of Fetal DNA Percentage

The extent of under- or overrepresentation of a particular 1-Mb bin in the maternal plasma is linearly correlated with the fetal DNA percentage (fetal %) in cases with fetal de novo copy number aberration involving that bin [4]. Hence, we calculated the fetal % based on the plasma genomic representations of the regions showing copy number aberrations in the test case using this equation:
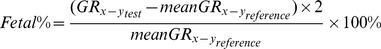



Only those 1-Mb bins which were completely covered by the region showing copy number aberration were included in the calculation of fetal %.

### Simulation Analyses

The sensitivity and specificity of detecting a microdeletion or a microduplication were affected by different parameters including the fetal % in the sample, the number of plasma DNA molecules sequenced and aligned, and the size of the aberration. Therefore, we performed computer simulation analyses to determine 1) the sensitivity of detecting a 3 Mb microdeletion/microduplication with the existing sequencing depth; and 2) the number of molecules needed to be analyzed to achieve a 95%/99% sensitivity when the fetal % was 5%.

This simulation represented an ideal situation when all analytical biases were minimized. In each simulation analysis, the whole genome (3,000 Mb) was divided into bins of equal size according to the desired resolution, which in the first instance was 3 Mb. For the detection of a subchromosomal aberration, we required three consecutive bins having genomic representation of >3 standard deviations (either over- or underrepresentation) away from the mean of the reference group in the same direction. Therefore, the bin size would be equal to 1/3 of the desired diagnostic resolution. For example, if we aim to detect aberrations of 3 Mb, the bin size would be 1 Mb. We assumed that the three bins covered by the microdeletion/microduplication would have an abnormal genomic representation resulting from the contribution of the minority population of fetal DNA. In the plasma, the expected proportion of total molecules (E) falling into a bin within an affected region can be calculated as:
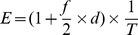
where f is the fetal DNA percentage in plasma,

d is the change in the chromosome number in the aberration (d equals to −1 for microdeletion and +1 for microduplication), and

T is the total number of bins for the whole genome

Simulations of 1,000 normal cases and 1,000 affected cases were performed assuming a binomial distribution of the plasma DNA molecules with the expected plasma representations as calculated above. The fetal %, the bin size and the total number of molecules being analyzed were changed to achieve the desired purpose. The simulation was conducted using the rbinom function in R (http://www.r-project.org/).

## Results

### Framework for Data Analysis

We used one lane of a flow cell on an Illumina HiSeq 2000 sequencer to analyze each maternal plasma sample of the six test cases and the eight controls. A mean of 211 million (range: 177 million to 236 million) DNA fragments were sequenced from each plasma DNA sample. Such sequencing resulted in a mean of 144 million (range: 96 million to 180 million) alignable and non-duplicated sequenced reads per case which was equivalent to 4.81 folds of the haploid human genome.

To obtain a plasma karyotype, the entire genome was divided into 2,687 1-Mb bins. We compared the genomic representation for each 1-Mb bin of the test sample with that of the reference group. For regions with normal genomic representation, the expected distributions of z-scores of all 1-Mb bins would be close to zero. We defined a reference interval as a z-score from +3 to −3. With such a reference interval, statistically approximately 0.3% of the bins would fall outside of this interval just by chance. As 2,687 bins were analyzed, we would on average expect that 8 bins would fall outside of the reference interval just by chance. To reduce false-positive calls, we therefore included an additional criterion of calling a copy number aberration only if three consecutive 1-Mb bins exhibited a z-score outside of the reference interval and in the same direction.

### Detection of Subchromosomal Copy Number Aberrations

**Figure 1:**
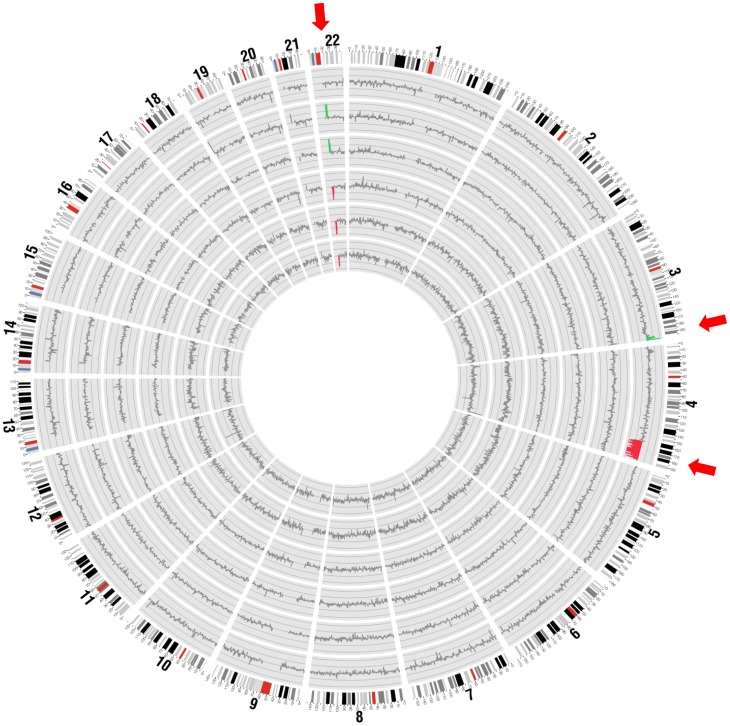
Circos plot of the detected copy number aberrations across the genome in maternal plasma. From inside to outside: cases 01 to 06. Chromosome ideograms (outermost ring) are oriented pter to qter in a clockwise direction. Each bar represents a 1-Mb window. Regions with three or more consecutive 1-Mb bins of increased or reduced representation in plasma are indicated by green and red bars, respectively. Red arrows highlight the approximate chromosomal locations on these aberrant regions.

The z-scores of all 1-Mb bins across the entire genome for each case were plotted using Circos plots [21] (Figure 1). In the test samples, 94.9%–98.7% of the 1-Mb bins showed normal representation. With the above-mentioned criterion of calling a copy number aberration only if three consecutive bins showed the same aberration, we correctly identified the copy number aberrations in all cases with no false-positives.

**Figure 2:**
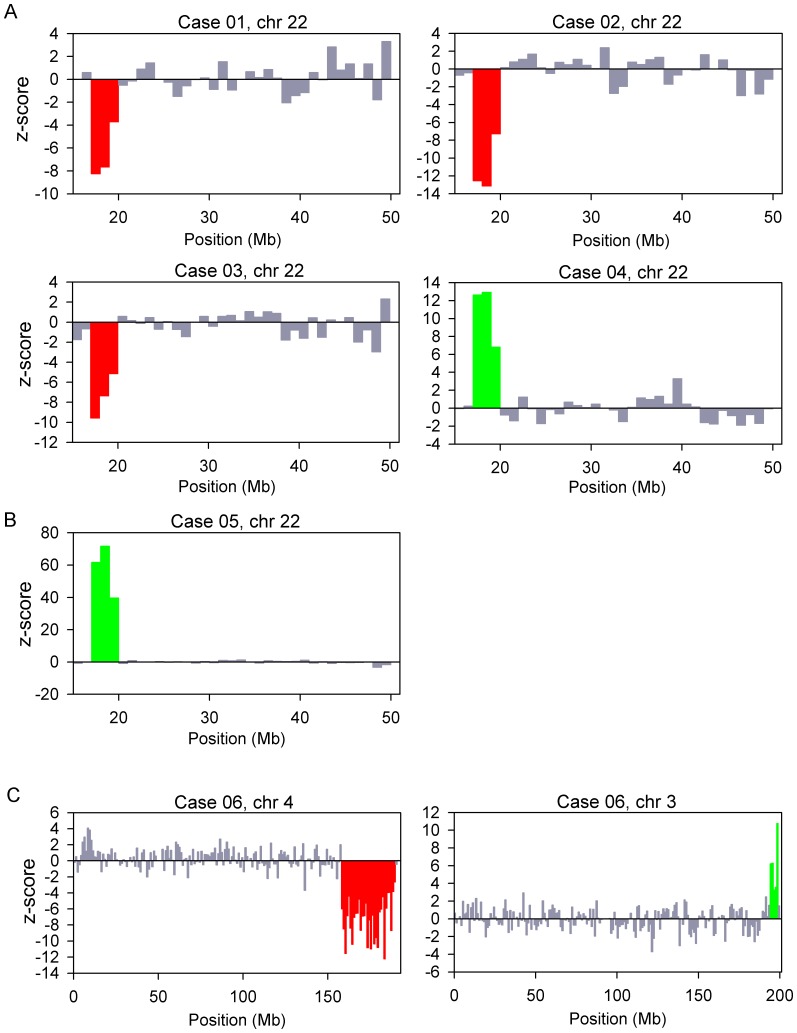
Copy number aberrations detected in maternal plasma. The chromosome(s) showing copy number aberrations for each case is shown. (A) Cases 01 to 04; (B) case 05; and (C) case 06. The genomic position is shown on the x-axis and the z-score is plotted on the y-axis. Each vertical bar represents a 1-Mb bin. Regions with three or more consecutive 1-Mb bins of increased or reduced representation in plasma are indicated by green and red bars, respectively.

Figure 2 shows the z-scores of all 1-Mb bins of the chromosome(s) showing copy number aberrations for each case. For cases 01, 02 and 03, we detected underrepresentation in three consecutive 1-Mb bins on the q arm of chromosome 22. These were the three cases with de novo 22q11.2 microdeletion. For cases 04 and 05, we detected overrepresentation in three consecutive 1-Mb bins on chromosome 22q. Case 04 was a case with a de novo 22q11.2 microduplication of 2.4 Mb. Case 05 was a case with a maternally-inherited microduplication in the same region. For case 05, since the mother herself harbored the microduplication, we could easily detect the aberration in the maternal plasma. This was supported by the extremely high z-score values (range, 39.7 to 71.7) for the three consecutive bins. Further exploration of noninvasive prenatal testing of the fetus could proceed with the use of SNP-based methods, namely relative mutation dosage or relative haplotype dosage analysis [10], [11], [22]. For case 06, we detected five consecutive 1-Mb bins with overrepresentation on the q arm of chromosome 3 and thirty-one consecutive 1-Mb bins with underrepresentation on the q arm of chromosome 4, which corresponded to a 5-Mb duplication on 3q and a 31-Mb deletion on 4q. For all cases, the copy number aberrations detected had sizes comparable to those confirmed by array CGH, FISH and/or QF-PCR. For case 05, the microduplication carried by the mother was confirmed by array CGH. For case 06, the balanced translocation carried by the mother was confirmed by full karyotyping.

### Fetal DNA Percentage

In this report, we used the DNA sequences from the regions showing under- or overrepresentation to estimate the fetal % in maternal plasma (Table 2). We validated this approach by comparing the fetal % calculated using this method and that using the chr Y-based method [4] for the three cases carrying male fetuses (i.e., cases 02, 03 and 04). The fetal % values agreed well between the two methods (Table 2). For the five cases with fetal de novo copy number aberrations, the fetal % ranged from 9.2% to 17.8%. For case 05, the fetal % estimated by the genomic representation of the microduplication was 96.7%, suggesting that almost all of the circulating DNA would harbor this change. This result is consistent with the fact that the mother carried the aberration.

**Table 2:** The fetal DNA percentage estimated by the alterations of the genomic representation of the regions affected by microdeletion/microduplication, and the proportions of chromosome Y sequences in the maternal plasma.

		Fetal DNA percentage	
Case	Fetal sex	By genomic representation of the affected chromosomal region(s)	By chr Y approach^a^
01	F	10.5%	−
02	M	17.4%	21.5%
03	M	9.2%	13.7%
04	M	17.8%	20.3%
05^b^	F	–	−
06	F	10.9%/13.4%^c^	−

a The chr Y approach is only applicable for those cases with a male fetus.

b For case 05, as the mother also carried the aberration, the genomic representation of the affected region in the maternal plasma could not be used to determine the fetal DNA percentage.

c The former and latter figures represent the fetal DNA percentage estimated from the microduplication on chromosome 3 and the microdeletion on chromosome 4, respectively.

### Simulation Analysis for Diagnostic Sensitivity

**Figure 3:**
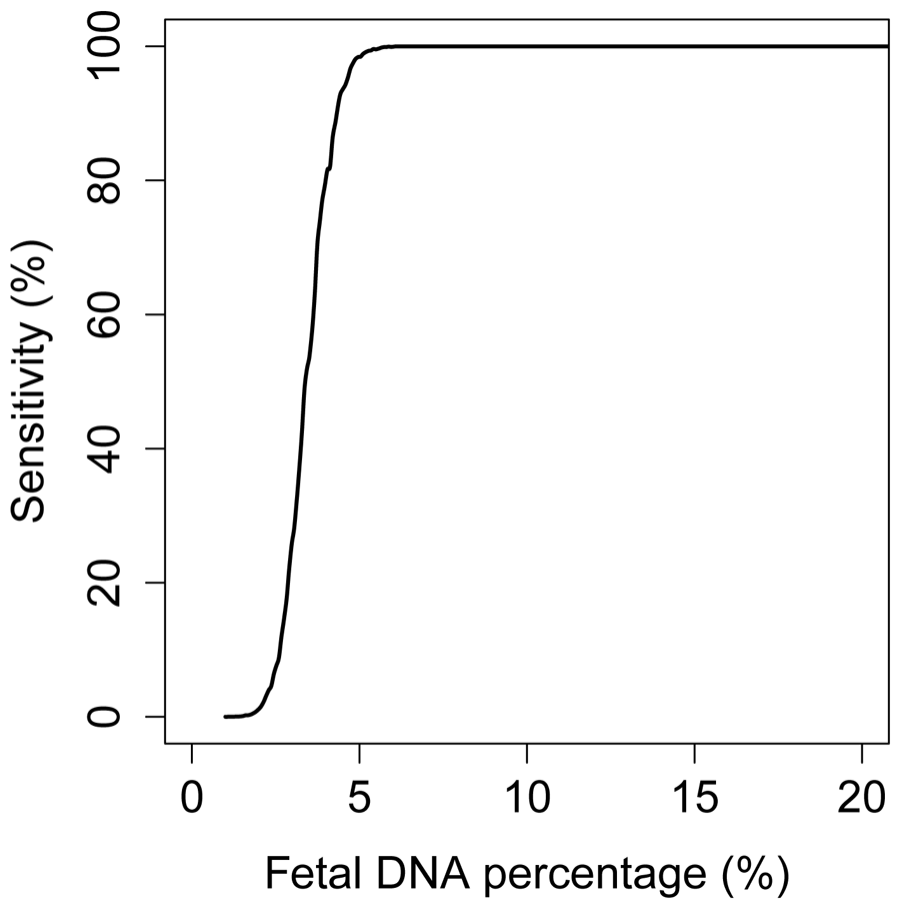
Diagnostic sensitivity for the detection of a 3 Mb microdeletion/microduplication. The diagnostic sensitivity for detecting the aberration is plotted against the fetal DNA percentage. The computer simulation analysis was performed assuming that a total of 150 million plasma DNA molecules were analyzed.

We used computer simulation to determine the diagnostic sensitivity of shotgun MPS-based noninvasive prenatal molecular karyotyping (Figure 3). With the existing sequencing depth of ∼150 million reads, the diagnostic sensitivity for detecting a 3 Mb chromosomal aberration would be approximately 96% when the fetal % is 5%. The sensitivity would increase to 99% when the fetal % reaches 6%. To detect chromosomal aberrations of smaller sizes, more plasma DNA molecules would need to be analyzed. Table 3 shows the number of plasma DNA molecules that needs to be analyzed to achieve 3 Mb, 2 Mb and 1 Mb diagnostic resolution with 95%/99% sensitivity, using the three consecutive bins criterion. To achieve a 95% diagnostic sensitivity, approximately 42,000 molecules in each bin would need to be analyzed. Thus, the total number of plasma DNA molecules that needs to be analyzed to detect a 2 Mb and a 1 Mb microdeletion/microduplication for a 95% diagnostic sensitivity would be 192 million and 380 million, respectively. To achieve a 99% diagnostic sensitivity, the total number of molecules that needs to be analyzed would be 240 million and 480 million for the two different resolutions, respectively.

**Table 3:** Number of molecules required to be sequenced and aligned to achieve different diagnostic resolutions and diagnostic sensitivities assuming that the fetal DNA percentage is 5%^a^.

Diagnostic sensitivity		Diagnostic resolution		
		3 Mb	2 Mb	1 Mb
95%	No. of molecules required in each bin	42,000	42,000	42,000
	Total no. of bins for the whole genome	3,000	4,500	9,000
	Total no. of molecules required for the whole genome	125 million	192 million	380 million
99%	No. of molecules required in each bin	53,000	53,000	53,000
	Total no. of bins for the whole genome	3,000	4,500	9,000
	Total no. of molecules required for the whole genome	160 million	240 million	480 million

a In this theoretical analysis, the diagnostic specificity is >99.9% for all cases based on the criteria that three consecutive bins having genomic representations >3SD (for either over- or underrepresentation) from the mean of the references in the same direction.

## Discussion

In this work, we have demonstrated the feasibility of performing the noninvasive prenatal detection of fetal chromosomal microdeletions and microduplications on a genomewide level and at 3-Mb resolution. We were able to detect fetus-derived subchromosomal deletions or duplications involving chromosomes 3q, 4q or 22q in 5 cases. In the sixth case, maternally-derived microduplication of chromosome 22q was detected, as evidenced by the very high z-scores seen. These results represent an important step forward compared with the previous reports by Peters et al [8] and Jensen et al [9] which were focused primarily on testing for copy number aberrations in one or a small number of genomic regions. Our data demonstrate that shotgun MPS can be used for detecting subchromosomal copy number aberrations on the genomewide scale, in other words, for obtaining a fetal molecular karyotype.

In three of the studied cases, maternal plasma samples were taken after invasive procedures. The fetal DNA percentages in these cases range from 9.2 to 17.4% which are within the range previously observed by Chiu et al [4] for samples collected prior to invasive procedures. Similarly, while most of the studied samples were taken beyond the 20^th^ week of gestation, the fetal DNA percentages of these cases are also largely overlapping with those of samples taken earlier in gestation. Nonetheless, it would be useful to validate these results in future, prospective, large-scale multicenter studies using samples collected prior to any invasive procedures in the first and early second trimesters.

Analytically, our diagnostic algorithm requires three consecutive bins with z-scores of all above +3 or all below −3 for detecting a subchromosomal copy number aberration. This algorithm requires a copy number aberration to be detectable over a contiguous stretch of approximately 3 Mb. Indeed, our data indicate that our algorithm was able to detect a copy number aberration of 2.4 Mb (cases 04 and 05).

The depth of sequencing that we had performed to reach such diagnostic resolution was much higher than that needed for trisomy testing. Thus, for each case, we performed sequencing in one lane of an Illumina HiSeq 2000 sequencer, compared with the 12-plex shotgun sequencing using the same sequencing platform that is performed by at least one commercial provider of trisomy testing. At the current depth of sequencing and its resultant diagnostic resolution of 3 Mb, the current protocol could cover approximately 20% of the known pathogenic copy number variants [23]. We have predicted that 240 million and 480 million plasma DNA molecules would need to be sequenced and aligned to extend the diagnostic resolution to 2 Mb and 1 Mb, respectively, with a 99% sensitivity. At these diagnostic resolutions, shotgun MPS of maternal plasma DNA would be expected to cover approximately 50% and 80%, respectively, of the known pathogenic copy number variants [23]. With a continual increase in throughput of massively parallel sequencers and the concomitant reduction in sequencing costs, it is likely that the costs associated with such sequencing depths will reach a level that would be acceptable to healthcare providers in a few years’ time. The amount of sequencing required by this approach is already a significant reduction over our previously reported fetus-derived single nucleotide variation detection method which was performed using billions of sequenced reads per sample [10]. Further reduction in costs could come from targeted sequencing of genomic regions harboring pathogenic copy number variants, similar to what has been achieved for fetus-derived single nucleotide variation detection from maternal plasma [24], [25]. Finally, the advent of single molecule sequencing would also be expected to further improve the diagnostic accuracy of this approach as amplification process, which might distort the genomic representation of the sequenced molecules, is not needed [26].

In summary, we have demonstrated that it might be feasible to obtain a noninvasive prenatal molecular karyotype by shotgun MPS of maternal plasma DNA. We have shown that our method can detect fetal de novo copy number changes, unbalanced translocations and maternal copy number changes. Future studies could be designed to address the efficacy of the present approach for detecting a wider spectrum of subchromosomal copy number changes. These results have further expanded the diagnostic spectrum of noninvasive prenatal diagnosis. In conclusion, methods based on MPS analysis of maternal plasma DNA have been developed for the prenatal detection of whole chromosome aneuploidies [3]–[7], subchromosomal copy number changes and fetal mutations for single gene disorders [10]. This array of noninvasive tests could in the first instance be applied for screening of fetal genomic and chromosomal abnormalities. Abnormalities revealed by the noninvasive maternal plasma DNA tests could be further confirmed by conventional invasive prenatal testing. Upon validation by large-scale prospective studies, it is envisioned that noninvasive maternal plasma DNA sequencing could provide prenatal assessment of a large spectrum of fetal genomic and chromosomal abnormalities and provide safer prenatal assessments.
